# An Always Correlated gene expression landscape for ovine skeletal muscle, lessons learnt from comparison with an “equivalent” bovine landscape

**DOI:** 10.1186/1756-0500-5-632

**Published:** 2012-11-13

**Authors:** Wei Sun, Nicholas J Hudson, Antonio Reverter, Ashley J Waardenberg, Ross L Tellam, Tony Vuocolo, Keren Byrne, Brian P Dalrymple

**Affiliations:** 1 Animal Science and Technology College, Yangzhou University, Yangzhou, 225009, China; 2 Food Futures Flagship, 306 Carmody Rd., St. Lucia, Brisbane, Queensland, 4067, Australia; 3 Livestock Industries, Commonwealth Scientific and Industrial Research Organisation, Queensland Bioscience Precinct, 306 Carmody Rd., St. Lucia, Brisbane, Queensland, 4067, Australia

## Abstract

**Background:**

We have recently described a method for the construction of an informative gene expression correlation landscape for a single tissue, longissimus muscle (LM) of cattle, using a small number (less than a hundred) of diverse samples. Does this approach facilitate interspecies comparison of networks?

**Findings:**

Using gene expression datasets from LM samples from a single postnatal time point for high and low muscling sheep, and from a developmental time course (prenatal to postnatal) for normal sheep and sheep exhibiting the Callipyge muscling phenotype gene expression correlations were calculated across subsets of the data comparable to the bovine analysis. An “Always Correlated” gene expression landscape was constructed by integrating the correlations from the subsets of data and was compared to the equivalent landscape for bovine LM muscle. Whilst at the high level apparently equivalent modules were identified in the two species, at the detailed level overlap between genes in the equivalent modules was limited and generally not significant. Indeed, only 395 genes and 18 edges were in common between the two landscapes.

**Conclusions:**

Since it is unlikely that the equivalent muscles of two closely related species are as different as this analysis suggests, within tissue gene expression correlations appear to be very sensitive to the samples chosen for their construction, compounded by the different platforms used. Thus users need to be very cautious in interpretation of the differences. In future experiments, attention will be required to ensure equivalent experimental designs and use cross-species gene expression platform to enable the identification of true differences between different species.

## Findings

The availability of gene expression datasets derived from the same tissue from animals with different genetic backgrounds, different developmental stages, and different environmental perturbations facilitates the construction of informative tissue specific gene expression correlation networks. The “Always Correlated” (AC) landscape approach provides a simple method for the construction of informative networks from relatively small datasets [1]. In particular the approach facilitates the identification of coherent modules of functionally related genes. The availability of equivalent tissue specific networks from different species would enable comparison between species for the same tissue and potentially the identification of common and/or species specific features.

### Constructing the ovine AC skeletal muscle transcriptional landscape and identification of modules

In order to construct the AC landscape for ovine LM muscle, we defined six groups of samples for the generation of individual condition gene expression correlation landscapes (Table 1). All RNA samples were from the LM muscle of sheep and were analyzed with the same GeneChip® Bovine Genome microarray (Affymetrix). The microarray contains 24,027 bovine probe sets representing ~19,000 UniGene clusters and 101 probe sets representing control elements. The probe sets on the microarray were annotated as previously described, using the UMD2.0 and Btau4.0 bovine genome assemblies [2]. The full annotation is provided in Additional file 1. Data acquisition criteria were as follows: firstly probe sets with a dubious gene assignment (for example with no or multiple genes predicted for the same probe set) were removed; secondly, for those genes represented by more than one probe set, the probe set with the highest expression level (averaged across all samples) was assigned to that gene. The edited data was normalized using MAS5 [3]. Genes with a “Present” flag at least one time point were retained for the next step in the analysis. Genes with no significant deviation of expression from the mean defined by one standard deviation across each dataset, or subset, were removed from the calculation of correlation coefficients to reduce spurious correlations.

**Table 1:** Sources of gene expression data contributing to the analysis groups

**Analysis group**	**Time points**^**1**^	**Number of arrays**^**2**^	**Number of genes in network**
Callipyge	80d^3^, 100d, 120d, T0^4^, P10d^5^, P20d, P30d, T12	16	4,176
Normal	80d, 100d, 120d, T0, P10d, P20d, P30d, T12	19	3,732
Prenatal (Callipyge and normal)	80d, 100d, 120d,	15	3,081
Postnatal (Callipyge and normal)	T0, P10d, P20d, P30d, T12	20	3,476
High-Low (high and low muscling phenotypes)	T78	40	3,462
Overall	80d, 100d, 120d, T0, P10d, P20d, P30d, T12, T78	75	17,308
AC landscape	intersection of above networks		1,661

^1^Data for 80d, 100d, 120d normal is from Byrne et al., 2010 [4], data for T0 and T12 is from Vuocolo et al., 2007 [5], data for 10d, 20d and 30d is from Fleming-Waddell et al., 2007 [6] and data from T78 is from Kogelman et al., [7].

^2^Each array was from a separate individual.

^3^Days post conception.

^4^Weeks post natal.

^5^Days post natal.

**Figure 1:**
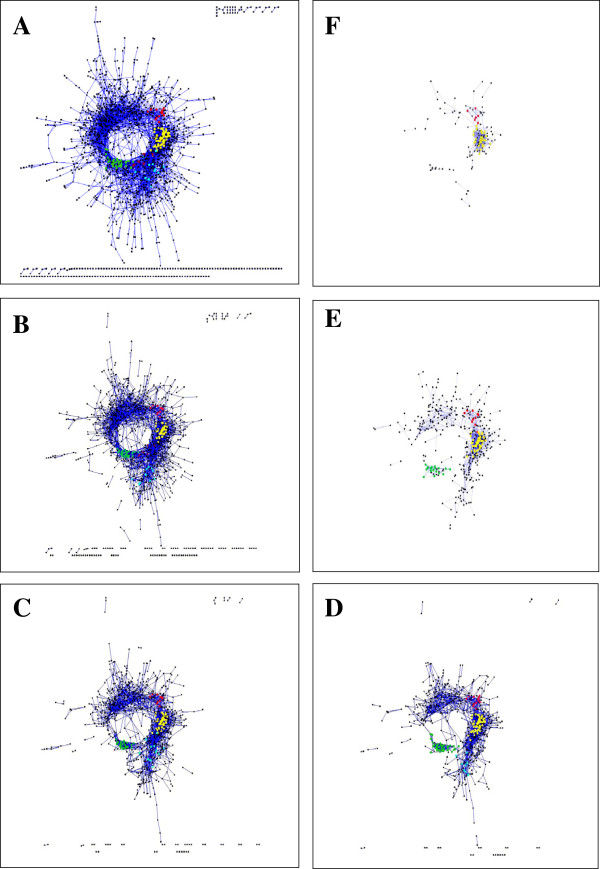
**AC landscape at different correlation coefficient cut-offs.A**) no cut off, **B**) 0.7, **C**) 0.8, **D**) 0.85, **E**) 0.9, **F**) 0.95. Modules are coloured as follows; red – muscle, yellow – mitochondria, green – ribosome, purple – translation, cyan – proteosome. The AC transcriptional landscape Cytoscape file is available in Additional file 2 .

The PCIT program [8] implemented as a package in R [9] was used to generate a total of six co-expression networks from the sets of gene correlation coefficients (Table 1). PCIT combines partial correlation coefficients with information theory to determine locally significant correlations automatically, avoiding the need for the specification of fixed correlation cut-offs. In order to facilitate the comparison between the ovine and bovine datasets the microarrays were grouped as closely as possible to the grouping of the bovine microarrays. The majority of the genes were present in the “Overall” network, whilst between three and just over four thousand genes were represented in the networks build from subsets of the data (Table 1). These networks were highly interconnected (contained a high ratio of edges to nodes), rendering the identification of modules within the network very hard (data not shown). The AC landscape was constructed by selecting those pairs of genes whose expression profiles were found to be significantly correlated by PCIT in all six networks. The final AC landscape comprised one large cohesive network (1,465 nodes) separate from a large number of very small networks containing two to four genes each, with a total landscape of 1,661 nodes and 5,196 edges (Figure 1A). Of the 5,196 edges, 1,368 (26.3%) represented negative correlations and 3,828 (73.6%) represented positive correlations.

To identify modules of genes within the sheep LM AC landscape a series of correlation coefficient based cut-offs were applied (Table 2 and Figure 1) and at each cut-off modules were identified using the Cytoscape MCODE plug-in [10] and annotated using the Cytoscape BiNGO plug-in [11] (Table 2). For each module, at each correlation cut-off, the MCODE parameters were gradually relaxed until the maximum size of each module was reached immediately preceding a major step increase of the modules’ connectivity into the surrounding network. In general the modules increased in size as the correlation cut-off was decreased from 0.95, and after reaching a maximum size, decreased in cohesiveness as the correlation cut-off was further decreased. Thus modules were defined using different correlation cut-offs, from >0.85 to >0.7. Five modules were present in the large, cohesive network: *muscle*, *mitochondrial proteins (nuclear encoded)*, *ribosomal proteins*, *regulation of ubiquitin ligation* and *translation* (Figure 1A, Additional file 2). A full listing of the genes in each module is provided in Additional file 3.

**Table 2:** Identification of functional modules in the AC landscape

**Module**	**Correlation cut-off**						**Description of the key GO term**	**GO enrichment P-value**
	**>0.95**	**>0.90**	**>0.85**	**>0.80**	**>0.70**	**none**		
Mitochondria	29^1^	39	**51**^2^	40	35	35	mitochondrial electron transport, NADH to ubiquinone	2.0370*10^2E-26^
Ribosome		18	**37**	23	29	33	translation	3.7793*10^3.8E-47^
Muscle	6	10	**12**	10	10	10	muscle contraction, muscle system process	3.0278*10^3E-8^
Regulation of ubiquitin-protein ligase activity			8	**13**	11	nd^3^	negative regulation of ubiquitin-protein ligase activity	1.2510*10^1.3E-5^
Translation					**6**	nd	translational elongation	1.1387*10^1.1E-8^

^1^ Number of genes in each module for each correlation cut-off of the “AC” landscape was calculated using the MCODE plug-in in Cytoscape. A list of genes in each module is included in Additional file 3.

^2^ Bold values have been taken as the optimal size of the module.

^3^ Module not detected.

### Comparison of the ovine and bovine AC landscapes

The ovine skeletal muscle co-expression landscape contains just under half the nodes, but ~60% more edges per node, and an eight times larger percentage of negative correlations than the cattle landscape [1]. The latter values were somewhat surprising, given that the two analyses started with a similar number of genes and achieving a similar ratio of edges to nodes in the two networks is predicted to lead to a very small ovine AC landscape. It is not clear whether these differences reflect the source of the samples used, the gene expression platform (in particular the use of a bovine microarray for analysis of the ovine samples), the quality of the gene expression data, or a combination of all the above.

There was a small, but significant (hypergeometric test >= 395, p-value = 9.2E-07), overlap of 395 genes between the two landscapes (Table 3). In addition to the intrinsic differences between bovine and sheep, the small overlap may reflect data quality, arising from the sampling and microarray platform differences. Like the bovine LM landscape [1] modules for mitochondrial proteins (nuclear encoded), ribosomal proteins and muscle/glycolysis were identified. However, in line with the low overlap in genes between the two networks there were relatively few genes in common between modules annotated with the same roles (Table 3). Similar to the bovine LM landscape, the genes encoding the muscle structural proteins were not highly clustered, but some small clusters of these genes were observed. The absence of strong clustering of the genes encoding muscle contractile structural proteins in both datasets suggests that even within a single muscle there is a less discrete, and more continuous, range of muscle fibre structural protein compositions at the anatomical level than might have been expected. This might also be in part due to the long developmental time series analysed, with relationships between contractile proteins changing with age of the animals.

**Table 3:** Overlap of the gene composition of modules in the ovine and bovine AC landscapes

**Ovine module name**	**Number of genes**			**Bovine module name**
	**Ovine only**	**Both**	**Bovine only**	
Mitochondrial	47	4	12	Mitochondria (nuclear encoded)
Ribosome	36	1	6	Ribosomal proteins
Translation	6	0	7	Ribosomal proteins
Muscle	8	4	54	Glycolysis/fast twitch
Full landscape	1266	395	3111	Full landscape
Full landscape – annotation overlap^1^	1138	395	2166	Full landscape – annotation overlap

^1^Genes that have been annotated in both landscapes.

In a more detailed comparison of the two landscapes we observed that only eighteen identical edges were present in both landscapes. Given that the module structure appeared to be conserved between the landscapes, but contained different genes, this may be due to the differences between the genes correlated within modules as a consequence of the phenotypes rather than sampling or microarray platform differences. However, if this was the result of platform related issues, this may have also led to different performances of the probes on the arrays for the same genes, equally impacting the correlations leading to the final network. Indeed, of the 14,041 genes confidently annotated on the Affymetrix microarray and 17,101 genes confidently annotated on the Agilent microarray only 11,712 genes could be confidently linked between the two datasets. It also appears that although the objective was to obtain a core network the design of the experiments still had an impact on the genes represented and the modules observed. For example, there was no “*cell cycle*” or “*fat*” module in the ovine network and no “*regulation of ubiquitin-protein ligase activity*” module in the bovine network, although genes from these modules were represented on the arrays and probes returned informative data.

### Muscle structural subunit genes in the ovine AC transcriptional landscape

**Figure 2:**
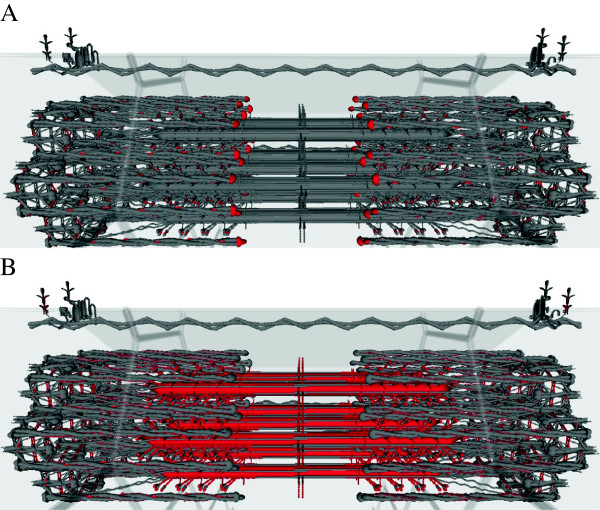
**Visualisation of data with VMus3D.A**) sheep. **B**) cow. Red indicates the presence of a gene coding a muscle structural protein in the “muscle module”. Grey indicates that the gene coding this protein was not present in the muscle module. The picture was generated using the virtual muscle 3D viewer [12] .

Of the twelve genes in the ovine muscle module, six encoded muscle structural proteins and five encoded enzymes involved in muscle metabolism (Table 4). The positions of the genes encoding muscle structural subunits in the rest of the ovine skeletal muscle AC landscape were determined (Table 4). Around half of the genes studied were present on the landscape, however except for the muscle module and adjacent to the module and a small cluster of genes adjacent to the mitochondrial module there was little clustering of genes encoding muscle structural proteins. In addition, apart from the cluster of fast twitch subunits adjacent to the mitochondrial module there was no separate clustering of fast and slow twitch fibre associated subunits. The data was mapped onto the Virtual Muscle 3D (VMus3D) [12] to determine if the products encoded by the genes in the muscle module were co-located in the macromolecular structure of muscle. No apparent clustering of products was observed (Figure 2). Perhaps not surprisingly genes encoding the costamere and z-disk proteins were not clustered with the genes encoding contractile proteins, consistent with many of the latter proteins also having roles in non-muscle structures and systems [12].

**Table 4:** Location of the genes encoding muscle structural protein subunits in the AC landscape

**Module name**	**Slow twitch fibres**^**1**^	**Fast twitch fibres**	**Both slow and fast twitch fibres**	**Fibre type specificity is not known**
Muscle	MYL2	TNNT3		TNNT1, FHL1, TMOD4, MYOZ1
Near *“muscle”*	TTN1	MYH2	ACTN2	TRIM54, TCAP, MYOT, DES, MYBPC1
Near *“mitochondrial”*		TNNC2, MYLPF, MYL1		SMPX
Near *“regulation of ubiquitin–protein ligase activity”*			**ACTA1**^2^	
Near * “translation” *		TPM1		
Elsewhere in AClandscape			**OBSCN**, NEB,	DMD, ANKRD1
Not in the AClandscape	MYL3, MYH7, TNNC1, TNNI1, TPM3	MYH1, MYBPC2, TNNI2,	MYBPH, ACTN3	MYOM2, MYOM3, MYBPC3, TNNI3, TNNT2, TPM2, MYPN, KBTBD10, KBTBD5, CSRP3, LMOD2, UNC45B, SGCA, CMYA5, PDLIM3, LRRC39, XIRP2, TRIM63

^1^Fibre type assignments of proteins are from [13], except for TMOD1 and TMOD4 [14] and MYL6B [15].

^2^Expression of genes encoding proteins in bold are negatively correlated with the majority of the members of the module.

### Potential impact of sample choice on AC modules

The final gene in the muscle module encoded FAF1, FAS-associated factor one, which is highly expressed in skeletal muscle [16]. Although probes for *FAF1* are present on the bovine Agilent array platform and return informative signals (consistent with expression in muscle contractile cells) *FAF1* was not present in the bovine AC landscape. FAF1 contains a ubiquitin-binding motif and has recently been reported to associate with the valosin-containing protein (VCP) purified from muscle, the resulting complex may interact transiently with the 26S proteosome [17]. Mutations in VCP cause inclusion body myopathies, it has been proposed that VCP plays a role in protein homeostasis, extracting proteins from protein complexes for degradation by the 26S proteosome [18] and that disruption of this role leads to accumulation of undegraded proteins [19]. The ubiquitin-dependent proteolytic system is the major proteolytic system in skeletal muscle [20]. The Callipyge mutation has been proposed to increase muscle mass through a reduction in the rate of muscle protein degradation, although this has been proposed to be through increased levels of calpastatin, rather than decreased activity of the 26S proteosome [21,22]. In addition, the proteosome was identified as a potential determinant of the muscling trait in the high-low muscling animals used in the analysis reported here [7]. Interestingly, no “*regulation of ubiquitin-protein ligase activity*” module was present in the bovine network, although genes from these modules were represented on the arrays and probes returned informative data. The ubiquitin-ligases play a role in the targeting of proteins to the 26S proteosome for degradation. This difference between the two AC landscapes is suggestive of the source of the samples influencing the resulting networks and that the comparison has potentially identified a difference related to a role of the 26S Proteosome in the Callipyge animals and more generally in high and low muscling phenotypes in sheep compared to another high muscling genotype, Myostatin deficiency [1], in cattle.

### Identification of putative key transcription factors

310 of the 898 transcription factors (TFs) analysed (see Hudson et al., 2009 [1]) were included in the ovine LM AC landscape, of which 4 were present in the modules (Table 5). “Module-to-regulator” relationships were computed based on the correlation values obtained from the “Overall” network and a number of known regulators of the functions/attributes determined for the modules were identified (Table 5). For example MEOX2 (*muscle* module) is involved in muscle development [23]. COPS5 (*mitochondrial* module), aka JAB1, is a component of the COP9 signalosome complex, which regulates the ubiquitin conjugation pathway [24], and was identified by both approaches. COPS5 has previously been shown to be involved in the regulation of the mitochondrial apoptotic pathway through specific interaction with BCL2L14 (aka BclGs) which is a regulator of mitochondrial apoptosis [25], YBX1 appears to have mismatch-repair activity in human mitochondria [26]. HIF1AN is involved in the regulation of HIF1 [27], which is involved in the regulation of the activity of mitochondria and it’s expression is also regulated by mitochondria via reactive oxygen species [28]. YY1 (*ribosomal* module) is one of small number of TFs with predicted binding sites in the promoter regions of many ribosomal protein genes [29].

**Table 5:** Assignment of Transcription Factors to robust modules

**Module**	**TFs in a module in the AC and identified by “Module-to-Regulator” analysis**	**TFs in a module in the AC landscape only**	**Top 10 TFs identified by the “Module-to-Regulator” analysis only**^**1**^
Muscle	none	none	KLF9, COPS5, HIF1AN, PREB, TCF7L2, SMARCA1, SMARCAD1, CHD1, CSDA, MEOX2 [23]
Mitochondrial	COPS5 [25]	none	SMARCAD1, CHD1, TCF7L2, **HIF1AN**^2^, SMARCA1, BPTF, PREB, MEOX2, YBX1 [26]
Ribosomal	none	none	BTF3, GTF2H5, CAMTA1, ZHX1, YY1 [29], BMI1, NR3C1, SUB1, ZBTB1, RBL2
Regulation of ubiquitin–protein ligase activity	BPTF	SUZ12	TCEB1, TAF9, COPS5 [30], SMAD5, SOX4 [31], JMJD1C, TCF4 [32], SMARCE1, NCOA1
Translation	BTF3	none	YBX1, TAF10, PHB2, ASH1L, TULP4, TBX3, RBM39, MLL3, RBL2

^1^ In descending order of strength of absolute average correlation coefficient. References providing experimental evidence supporting our computational output are provided.

^2^TFs also identified in the presumed equivalent module in the bovine AC landscape are in bold.

For the “*regulation of ubiquitin-protein ligaseactivity*” module, SUZ12 has a GO annotation for “histone ubiquination”. TCEB1 has a GO annotation for “ubiquitin-ligase complex” and “ubiquitin-dependent protein catabolic process”. TAF9 has a GO annotation of “regulation of proteosomal ubiquitin-dependant protein catabolic process”. In addition, COPS5 regulates exosomal protein deubiquitination and sorting [30], SOX4 interacts with ubiquitin-conjugating enzyme 9 (UBC9), which represses the transcriptional activity of SOX4 [31], and TCF4 regulates the expression of ubiquitin c-terminal hydrolase L1 (UCHL1) [32]. Thus, of the 11 proteins encoded by genes identified by the “Module-to-Regulator” analysis, six have a link with processes involving ubiquitin. In contrast, for example only 1 of the proteins identified in the analysis for the *translation* module is annotated with a GO term which includes the word ubiquitin.

However, even though the analyses in the two species both appear to correctly identify some TFs involved in the regulation of the function of the module there is only one gene, *HIF1AN*, in the overlap between the transcriptional regulators identified in the ovine and bovine “Module-to-Regulator” analyses (Table 5). Again it is likely that the experiment specific factors described above have contributed to this small overlap, which is not significant (hypergeometric test of an overlap of one gene in the *mitochondrial* module p-value = 0.23), and that a significant rate of false positives may be generated using these methods.

## Conclusions

Despite apparent similarities between the datasets, a development time course overlaid with a muscle growth contrast, the differences in the composition of the experimental samples and design appears to have significantly impacted the final landscapes generated using the AC approach. The detection of true differences between cattle and sheep LM muscle awaits the availability of appropriately generated orthologous datasets using, for example, transcript sequencing techniques from as close as possible equivalent experiments. However, generating a truly orthologous dataset between two different species, even for equivalent tissues, with equivalent analysis parameters may not be a trivial process.

### Availability of supporting data

With the exception of the data for the 80d, 100d and 120d LM muscle sheep callipyge genotype samples which is unpublished (Personal communication RL Tellam, K Byrne, T Vuocolo and N Cockett), the sheep gene expression data sets supporting the results of this article are available in the NCBI GEO repository, GSE5195 (10d, 20d, 30d, LM muscle sheep, callipyge and normal genotypes), GSE5955 (T0 and T12 LD muscle sheep, callipyge and normal genotypes), GSE20112 (80d, 100d, 120d LM muscle sheep normal genotypes), GSE20552 (T78, LM muscle sheep, High-Low).

The other data sets supporting the results of this article are included within the article and its additional files.

## Competing interests

The author(s) declare that they have no competing interests.

## Authors’ contributions

BD conceived the study, BD, NH, TR and WS designed the analyses, WS AJW and BD undertook the analyses, BD, NH, WS, AJW, TR wrote the manuscript and RT edited the manuscript. RT, KB and TV provided unpublished gene expression data. All the authors read and approved the final version.

## Supplementary Material

Additional file 1 Bovine Affymetrix probeset annotation file. Click here for file

Additional file 2 Sheep AC transcriptional landscape Cytoscape session file. Click here for file

Additional file 3 List of genes in each module in the sheep skeletal muscle AC gene expression landscape. Click here for file
